# The Ground-Set-Cost Budgeted Maximum Coverage Problem

**DOI:** 10.1007/s00224-025-10248-5

**Published:** 2025-11-20

**Authors:** Irving van Heuven van Staereling, Bart de Keijzer, Guido Schäfer

**Affiliations:** 1 Networks & Optimization Group, Centrum Wiskunde & Informatica, Science Park 123, Amsterdam, 1098 XG The Netherlands; 2 Department of Informatics, King’s College London, 30 Aldwych, London, WC2B 4BG United Kingdom; 3 Institute for Logic, Language and Computation, University of Amsterdam, Science Park 107, 1098 XG Amsterdam, The Netherlands

**Keywords:** Maximum coverage problem, Approximation algorithms, Hypergraphs, Submodular optimization, Sponsored search

## Abstract

We study the following natural variant of the budgeted maximum coverage problem: We are given a budget *B* and a hypergraph $G = (V, E)$, where each vertex has a non-negative cost and a non-negative profit. The goal is to select a set of hyperedges $T \subseteq E$ such that the total cost of the vertices covered by *T* is at most *B* and the total profit of all covered vertices is maximized. This is a natural generalization of the maximum coverage problem. Our interest in this problem stems from its application to bid optimization in sponsored search auctions. It is easily seen that this problem is at least as hard as budgeted maximum coverage (where the costs are associated with the selected hyperedges instead of the covered vertices). This implies $(1-1/e+\epsilon )$-inapproximability for any $\epsilon> 0$. Furthermore, standard greedy approaches do not yield constant factor approximations for our variant of the problem. In fact, through a reduction from Densest *k*-Subgraph, it can be established that our problem is inapproximable up to a constant factor, conditional on the exponential time hypothesis. Our main results are as follows: (i.) We obtain a $(1 - 1/\sqrt{e})/2$-approximation algorithm for graphs. (ii.) We derive a fully polynomial-time approximation scheme (FPTAS) if the incidence graph of the hypergraph is a forest (i.e., the hypergraph is *Berge-acyclic*). We extend this result to incidence graphs with a fixed-size feedback hyperedge node set. (iii.) We give a $(1-\varepsilon )/(2d^2)$-approximation algorithm for all $\varepsilon> 0$, where *d* is the maximum vertex degree.

## Introduction

In the *budgeted maximum coverage problem* we are given a hypergraph $G = (V, E)$ with a non-negative cost $c(e) \in \mathbb {R}_{\ge 0}$ for every hyperedge $e \in E$ and a non-negative profit $p(i) \in \mathbb {R}_{\ge 0}$ for every vertex $i \in V$, and a non-negative budget $B \in \mathbb {R}_{\ge 0}$. The goal is to select a set of hyperedges $T \subseteq E$ of which the total cost is at most *B* such that the total profit of all vertices covered by the hyperedges in *T* is maximized.

This is a fundamental combinatorial optimization problem with many applications in resource allocation, job scheduling and facility location (see, e.g., [1] for examples). Feige [2] showed that this problem is not polynomial-time approximable within a factor of $(1-1/e + \epsilon )$ for any $\epsilon> 0$ unless $\textsf{P} = \textsf{NP}$, even if all hyperedges have unit cost. Khuller, Moss and Naor [3] derived a $(1-1/e)$-approximation algorithm for the budgeted maximum coverage problem (which is the best possible). Their algorithms are based on a natural greedy approach in combination with a standard enumeration technique. Similar approaches were used to derive constant factor approximation algorithms for several other variants and generalizations of the maximum coverage problem.

In this paper, we study the following natural variant of the budgeted maximum coverage problem, which we call the *ground-set-cost budgeted maximum coverage problem (GBMC)*: We are given a hypergraph $G = (V,E)$ with a non-negative cost $c(i) \in \mathbb {R}_{\ge 0}$ and a non-negative profit $p(i) \in \mathbb {R}_{\ge 0}$ for every vertex $i \in V$, and a non-negative budget $B \in \mathbb {R}_{\ge 0}$. For a subset $T \subseteq E$, define $c(T) = \sum _{i \in \cup T} c(i)$ and $p(T) = \sum _{i \in \cup T} p(i)$ as the total cost and profit, respectively, of all vertices covered by the hyperedges in *T*.^1^ Our goal is to select a set of hyperedges $T \subseteq E$ such that the total cost *c*(*T*) of all covered vertices is at most *B* and the total profit *p*(*T*) of all covered vertices is maximized. To the best of our knowledge, this problem has not been studied before.

Note that a crucial difference here is that in our problem costs are incurred per covered vertex, while in the budgeted maximum coverage problem costs are incurred per selected hyperedge. Albeit seemingly minor, this change makes the problem much harder to tackle algorithmically. More specifically, most greedy approaches (which give rise to constant factor approximation guarantees for several variants of the maximum coverage problem) turn out to be inapplicable in our setting because of the following reason: The basic idea underlying these greedy approaches is to select in each iteration a hyperedge that is most *cost-efficient*, i.e., maximizes the ratio of the profit of newly covered vertices over the cost of selecting the hyperedge. A property that is crucially exploited in the analysis of these algorithms is that the cost for selecting a hyperedge is constant, i.e., its cost-efficiency can only decrease throughout the course of the algorithm (as more of its vertices get covered). However, this monotonicity property is no longer guaranteed in our setting because the cost for picking a hyperedge depends on the set of already covered vertices. In fact, it is not hard to see that in GBMC, the cost-efficiency of a hyperedge can either (abitrarily) increase or decrease from one iteration to the next, whereas in classical budgeted maximum coverage, the cost-efficiency can only decrease.

### Example 1

The following simple instance of GBMC shows that the approach of greedily selecting hyperedges according to highest cost-efficiency does not perform well, even when the hypergraph is a graph. Consider an instance with a budget of $B> 1$ and a graph of four vertices $\{1,2,3,4\}$, and three edges $\{\{1,3\},\{1,4\},\{2,3\}\}$. Vertices 1 and 2 both have profit 0, vertex 3 has profit 1, and vertex 4 has profit $B-1$. As for the cost, vertex 1 has cost *B*, vertex 2 has cost 1, and vertices 3 and 4 both have cost 0.

The greedy procedure would first select edge $\{2,3\}$ as its cost-efficiency is $(0+1)/(1+0) = 1$ (whereas for the remaining edges this would be lower: $\{1,3\}$ has cost-efficiency $(0+1)/(B + 0) = 1/B$, and $\{1,4\}$ has cost-efficiency $(0 + (B-1))/(B + 0) < 1$). After this first pick, no further edge can be selected because both remaining edges contain vertex 1, which has a cost equal to the full budget. The resulting solution thus has a total profit of 1, whereas the optimal solution would consist of selecting edges $\{\{1,3\},\{1,4\}\}$, and yield a profit of *B*.

Our motivation for investigating the vertex-cost budgeted maximum coverage problem is two-fold: (i) It is a generalization of the well-studied maximum coverage problem and a natural variant of the budgeted maximum coverage problem. (This holds because we can view maximum coverage as a special case of our problem where for each hyperedge there is a “dedicated” vertex that is contained in only *h*, which has a cost that corresponds to the cost of *h*. See also the reduction in the proof of Theorem 2 below.) (ii) It is a fundamental combinatorial optimization problem having several applications in practice. Of particular importance is its relation to the problem of computing optimal bids in sponsored search auctions such as Google Ads, which we describe next.

### Relation to Bid Optimization in Sponsored Search Auctions

An application for the problem studied in this paper is an optimization problem revolving around sponsored search auctions, such as Google Ads. In this section, we argue that the two problems are equivalent.

A typical sponsored search auction works as follows: Advertisers show their advertisements to users of search engines. To this aim, they bid on so-called *keywords* that are relevant to the queries that the users search for. When a user inputs a search query, an auction takes place among all advertisers that have bid on a keyword that is relevant to this query, and the advertisements of the winners of the auction are displayed to the user who entered the query. If the user clicks on an advertisement, the respective advertiser pays a certain amount of money to the search engine.

An advertiser has only a limited budget to spend on his online advertising campaign (typically on a daily basis). The objective is therefore to maximize profit (usually the expected number of clicks) by placing bids on the keywords in the best possible way. This optimization problem that the advertiser faces in this setting was studied extensively, see [4–7].

A crucial property of sponsored search auctions is that it is not allowed to bid on each query separately. Instead, an advertiser places bids on the keywords and as a result might specify multiple “conflicting” bids on keywords that are relevant to the same query. Sponsored search auctions resolve this by defining the *participating bid* of a query as the largest bid among all relevant keywords.
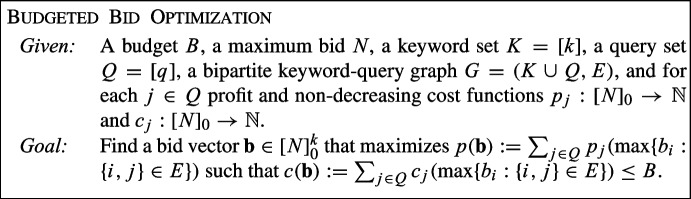


**Fig. 1:**
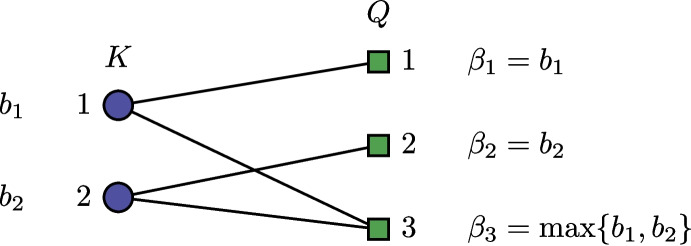
Example of a keyword-query graph with the respective participating bids

The Budgeted Bid Optimization problem that arises from this can thus be formulated as follows. We are given a set of keywords *K*, a set of queries *Q* and a bipartite keyword-query graph $G = (K \cup Q, E)$ that specifies which keywords are relevant to which queries. Each query $j \in Q$ has a cost $c_j$ and $p_j$, which depends on the participating bid of *j*. The goal is to specify a bid $b_i$ for every keyword $i \in K$ such that the overall profit is maximized while the total cost does not exceed a given budget *B*. See Fig. 1 for a simple example with only 2 keywords and 3 queries.

Feldman et al. [5] were the first to study this problem, which we will refer to as Budgeted Bid Optimization. They show that the problem is strongly $\textsf{NP}$-hard and that a weighted variant is inapproximable within a factor of $1-1/e + \epsilon$ for any $\epsilon> 0$. They derive a $(1-1/e)$-approximation for an easier variant where the budget constraint needs to be fulfilled in expectation.

Clearly, GBMC is a special case of Budgeted Bid Optimization, namely the case where one can only bid either 0 or 1 on each keyword (the hyperedges), while the queries correspond to the vertices with one profit/cost data point. However, we will show that the problems are equivalent, by providing an approximation-preserving reduction from GBMC to Budgeted Bid Optimization. This simplifies the analysis of approximation algorithms for Budgeted Bid Optimization, and implies that the results presented in this paper can be used for a larger variety of submodular optimization problems.

### Theorem 1

There is an approximation-preserving reduction from Budgeted Bid Optimization to GBMC.

The proof of Theorem 1 can be found in Appendix A.

### Our Contributions

The contributions presented in this paper are as follows:

1. We obtain a $(1 - 1/\sqrt{e})/2$-approximation algorithm for graphs (Section 2). The main idea here is to reduce this problem to the budgeted maximum coverage problem with an exponential number of hyperedges. However, we do not need to generate the exponentially large instance explicitly; but instead we make use of a concise representation of the instance and show that such instances can be approximated in polynomial time, given that we have access to an oracle that can select in polynomial time a hyperedge with approximately highest profit per unit of cost. As a last step in our reduction, we prove that such an oracle exists.
2. We derive in Section 3 a pseudo-polynomial time algorithm for the case when the incidence graph of the hypergraph is a forest (i.e., the hypergraph is *Berge-acyclic*). Further, we adapt this algorithm into a fully polynomial-time approximation scheme (FPTAS). At the core of this algorithm lies a bi-level dynamic program. The case of forests is important in its own right and, additionally, this algorithm constitutes an important building block of our $\mathcal {O}(1/d^2)$-approximation algorithm (see Contribution 4).
3. In Section 4, we extend the above algorithm to a pseudo-polynomial time algorithm for incidence graphs with a bounded set of nodes that covers all cycles (i.e., the general case, but parametrized). More specifically, we show that for any incidence graph with a fixed-size *feedback hyperedge node set*, i.e., a hyperedge node set such that removing it from the incidence graph leaves no cycles, there exists a pseudo-polynomial time algorithm for the GBMC problem.
4. We give a $(1-\varepsilon )/(2d^2)$-approximation algorithm for every $\varepsilon> 0$ for the general case, where *d* is the maximum degree of a vertex in the hypergraph (Section 5). In this algorithm, we first decompose the incidence graph of the hypergraph into a collection of at most *d* trees for which we compute an approximate solution by using our FPTAS for forests above. From this we then extract a solution that is feasible for the original instance and guarantees an approximation ratio of at least $(1-\varepsilon )/(2d^2)$.

Lastly, we note in Section 6 that for the general case of this problem, it seems very unlikely that a constant approximation factor can be achieved for this problem through a polynomial time algorithm: Assuming the exponential time hypothesis, through a reduction from Densest *k*-Subgraph, it can be established that even for the case of 3-uniform hypergraphs, no approximation factor better than $n^{{1/(\log \log n})^c}$ can be guaranteed in polynomial time, for some constant *c*.

With respect to the significance of the our results, our constant approximation factor achieved by Contribution 1 in the above list of contributions complements the inapproximability for the case of 3-regular hypergraphs, and highlights a dichotomy in approximability as a function of the size of the hyperedges in a GBMC-instance. Furthermore, besides that the graph case of our problem is a natural combinatorial restriction of the general GBMC problem, it corresponds (through Theorem 1) to a natural basic variant of the Budgeted Bid Optimization problem where there are only two bidding levels (i.e., “high” and “low” bids). Contributions 2 to 4 yield polynomial time algorithms with respect to fixing either of two distinct parameters of GBMC: The maximum degree *d* of a vertex in the hypergraph and the maximum size of feedback hyperedge node set in the incidence graph. When either of these parameters are low in value, these algorithms may be used to solve instances of the GBMC problem.

### Related Work

Much literature is available on the maximum coverage problem and its variants (see, e.g., [3, 8, 9] and the references therein). Most related to our problem is the budgeted maximum coverage problem [3]. As outlined above, the greedy approach of [3] cannot take into account that the costs are incurred per vertex instead of per set. Moreover, in [9], a generalized version of the budgeted maximum coverage problem is studied, but this generalization does not include GBMC as a special case.

Note that our GBMC problem on graphs reduces to the knapsack problem if the incidence graph is a matching. This problem is known to be weakly $\textsf{NP}$-hard and admits an FPTAS (see, e.g., [10]).

As outlined in Section 1, our GBMC problem is related to the *budgeted bid optimization problem*. This problem was first proposed in the paper by Feldman et al. [5]. The authors derive a $(1-1/e)$-approximation algorithm if the budget constraint is *soft*, i.e., has to be met in expectation only. In contrast, in the budgeted bid optimization problem considered here, this budget constraint is hard.

The GBMC problem can be seen as a special case of a more general set of problems where we have to maximize a submodular profit function subject to the constraint that a submodular cost function does not exceed a given budget. This can be seen by considering the set of hyperedges to be the ground set of the submodular functions. However, when we have oracle access to both submodular functions, it has been shown that this more general problem is not approximable within a factor of $\log (m)/\sqrt{m}$, where *m* is the number of elements in the ground set. This holds even for the special case that the objective function is the modular function that returns the cardinality of the set. This follows from Theorem 4.2 in [11]; see also [12].

### Preliminaries

For an integer $a \in \mathbb {N}$, we write [*a*] and $[a]_0$ to denote the sets $\{1, \ldots , a\}$ and $\{0, 1, \ldots , a\}$ respectively. When *F* is a family of sets, we write $\cup F$ to denote the set $\cup _{S \in F} S$.

Let $G = (V, E)$ be a hypergraph. The *incidence graph* *I*(*G*) of *G* is defined as the bipartite graph $I(G) = (E \cup V, H)$ with $H = \{\{e, v\}\ :\ v \in e\}$. We say that *G* is *acyclic* iff its incidence graph *I*(*G*) does not contain a cycle. Given a subset $E' \subseteq E$, we use $G[E']$ to refer to the *subgraph* of *G* induced by the hyperedges in $E'$, i.e., $G[E'] = (V', E')$ with $V' = \cup E'$. A hypergraph *T* is called a *subtree* of *G* iff *T* is a subgraph of *G* that is acyclic. For the special case where *G* is a graph, we say that *G* is a *star* iff all its edges intersect in a single vertex.

Throughout this paper we will use the convention that when discussing a hypergraph, *n* denotes the number of vertices of the hypergraph and *m* denotes the number of hyperedges of the hypergraph. Moreover, in the remainder of this paper, we assume without loss of generality that all costs (on the nodes or edges) are strictly positive.

As a preliminary result, we first note that GBMC cannot be approximated to within a factor of $1 - 1/e + \epsilon$ in polynomial time, for any $\epsilon> 0$, unless $\textsf{P} = \textsf{NP}$.

### Theorem 2

The GBMC problem cannot be approximated to within a factor of $1-1/e + \epsilon$ for any $\epsilon>0$ in polynomial time, unless $\textsf{P} = \textsf{NP}$.

### Proof

We use a reduction from the *maximum coverage problem*: We are given a hypergraph $G = (V, E)$ and a parameter $k> 0$. The goal is to choose a set of at most *k* hyperedges $X \subseteq E$ such that the number of covered elements $\left| \cup X\right|$ is maximized. This problem cannot be approximated within $1-1/e + \epsilon$ for any $\epsilon> 0$ unless $\textsf{P} = \textsf{NP}$ [2].

Given an instance $I = (G = (V,E), k)$ of the maximum coverage problem, we construct an instance $I' = (G' = (V',E'), c', p', B')$ of the GBMC problem as follows: Let the set of hyperedges in of *G* be $E = \{e_1, \dots , e_m\}$. Define $V' = V \cup \{w_1, \ldots w_{m}\}$, where $w_1, \ldots , w_{m}$ are new vertices that are not in *V*. Furthermore, define $E' = \{e_1 \cup \{w_1\}, \ldots , e_m \cup \{w_m\}\}$, $B' = k$, $c'(w_i) = 1$ for all $i \in [m]$ and $c'(v) = 0$ for all $v \in V$. Lastly, set $p'(w_i) = 0$ for all $i \in [m]$ and $p'(v) = 1$ for all $v \in V$.

Now, consider the bijection *f* between *E* and $E'$ that maps hyperedge $e_i \in E$ to the hyperedge $e_i \cup \{w_i\} \in E'$. It is easy to verify that for each subset $X \subseteq E$, the set $X' = \{f(e) : e \in X\}$ satisfies $c(X') \le B$ if and only if $|X| \le k$. Furthermore, $p'(X') = |X|$. Thus, *I* can be approximated within a factor of $1 - \frac{1}{e}$ if and only $I'$ can be approximated within a factor of $1 - \frac{1}{e}$, which proves the claim. $\square$

Note that in Section 6, we include a proof that the problem is also not even constant-factor approximable, when assuming the exponential time hypothesis (which is stronger than assuming $\textsf{P} \not = \textsf{NP}$).

## GBMC on Graphs

We study in this section the special case of GBMC where the hypergraph is a graph, i.e., the hyperedges all have size 2. We prove for this case the following main result.

### Theorem 3

There exists a polynomial-time $(1-1/\sqrt{e})/2$-approximation algorithm for the GBMC problem when the hypergraph is a graph.

To prove this, we analyse first a variant of the classical budgeted maximum coverage problem, where we have a type of oracle access to the problem instance (rather than direct access to the description of the instance). After designing an algorithm for this auxiliary problem in Section 2.1, we provide the proof of Theorem 3 in Section 2.2.

### Budgeted Maximum Coverage with Oracles

We consider now the classical budgeted maximum coverage problem. The result presented in this subsection will serve as a building block in the approximation algorithm presented in the next section, for solving GBMC on graphs.

A polynomial-time $(1-1/e)$-approximation algorithm for the budgeted maximum coverage problem was previously given in [3]. In the same paper, various simpler algorithms with worse approximation factors are presented. In this section, we present a variation of one of these algorithms that achieves a $(1-1/e)/2$-approximation guarantee, which can run even if the algorithm is not granted direct access to the input instance. We make this precise in the following definition.

#### Definition 1

(cost-efficiency oracle) Let $I = (G = (V,E),c,p,B)$ be an instance of the budgeted maximum coverage problem. For $\alpha \in [0,1]$, an $\alpha$*-approximate cost-efficiency oracle* for *I* is a function $f_I : 2^V \rightarrow E$ that maps a set of vertices $S \subseteq V$ to a hyperedge $e \in E$ such that $c(e) \le B$ and

$$\begin{aligned} \sum _{i \in e \setminus S} \frac{p(i)}{c(e)} \ge \alpha \cdot \sum _{i \in e' \setminus S} \frac{p(i)}{c(e')}. \end{aligned}$$

for all $e' \in E$ with $c(e') \le B$.

Thus, a cost-efficiency oracle takes as input vertex set *S* and selects the hyperedge with the approximately highest cost-efficiency (up to a factor $\alpha$), excluding the profit that would be contributed by vertices in *S*. Only hyperedges of which the cost does not exceed the budget are considered.

Let $I = (G = (V,E), c, p, B)$ be an instance of the budgeted maximum coverage problem, and let $f_I$ be an $\alpha$-approximate cost-efficiency oracle for this instance for some $\alpha \in (0,1]$. Consider now the following greedy algorithm $\mathcal {A}$ that takes as input only the cost-efficiency oracle $f_I$.

1. Set $S := \varnothing$ and $X := \varnothing$. Throughout the execution of the algorithm, *X* represents a feasible solution and *S* represents the set of vertices covered by *X*.
2. Let $e := f_I(S)$. If $S = V$ (i.e., there is no profitable hyperedge left) or if $c(e) + \sum _{e' \in X} c(e')> B$ (i.e., adding the hyperedge to *X* would exceed the budget), go to Step 3. Otherwise, set $X := X \cup \{e\}$, set $S = \bigcup X$, and repeat this step.
3. Output the solution with the highest total profit among the two solutions *X* and $\{e\}$.

#### Theorem 4

Algorithm $\mathcal {A}$ outputs an $(1-1/e^{\alpha })/2$-approximate solution to *I* in time $\mathcal {O}(n \cdot t)$, where *t* is the amount of time it takes to evaluate $f_I$.

The approximation factor is obtained by following rather closely the analysis given in [3] for a similar algorithm (that works without oracle access), hence we defer the proofs of Lemmas 5 to 8 to Appendix B. We essentially generalize the proofs in [3] by taking into account the additional factor $\alpha$, and the oracle-access assumption.

Let *k* be an iteration of Step 2 of algorithm $\mathcal {A}$ running on *I*, let $e_k$ be the edge that the $\alpha$-approximate cost-efficiency oracle returns at the *k*th iteration of Step 2. Let $T_0 = \varnothing$ and inductively define $T_k$ to be the set $T_{k-1} \cup \{e_k\}$, i.e., $T_k = \{e_1, \ldots , e_k\}$. Likewise, let $S_i = \cup T_i$, and let $S_0 = \varnothing$. Denote by $T^*$ an optimal solution to *I*. Note that if $\ell$ is the total number of iterations of Step 2, then in Step 3 the solutions $T_{\ell - 1}$ and $e_{\ell }$ are considered. Moreover, note that $T_{\ell }$ is typically not a feasible solution, as its cost may exceed *B*.

The following three lemmas provide us with useful inequalities that we need in order to prove Theorem 4.

#### Lemma 5

For each iteration *k* of Step 2 of algorithm $\mathcal {A}$, it holds that

$$\begin{aligned} \sum _{i \in (\cup T^*) \setminus S_{k-1}} p(i) \le \frac{B}{\alpha c(e_k)}(p(T_k) - p(T_{k-1})). \end{aligned}$$

#### Lemma 6

For each iteration *k* of Step 2 of algorithm $\mathcal {A}$, it holds that

$$\begin{aligned} p(T_k) - p(T_{k-1}) \ge \frac{\alpha c(e_k)}{B}(p(T^*) - p(T_{k-1})). \end{aligned}$$

#### Lemma 7

For each iteration *k* of Step 2 of algorithm $\mathcal {A}$, it holds that

$$\begin{aligned} p(T_k) \ge p(T^*) \left( 1 - \prod _{k' = 1}^k \left( 1 - \frac{\alpha c(e_{k'})}{B}\right) \right) . \end{aligned}$$

Lastly, for Theorem 4, we need the following basic insight on a specific function minimization problem.

#### Lemma 8

Let $a \in \mathbb {R}_{\ge 0}$ be any nonnegative real number and $n \in \mathbb {N}_{>1}$ be a positive natural number. The function $g(x_1, \ldots , x_n) = 1 - \prod _{i = 1}^n\left( 1 - \frac{x_i}{B}\right)$ on the domain $D = \{x \in \mathbb {R}^n : \sum _{i = 1}^n x_i = a\}$ achieves its minimum at the point where $x_i = a/n$ for all $i \in [n]$.

With the above four lemmas, we are ready to prove the claimed approximation factor for Algorithm $\mathcal {A}$.

#### Proof of Theorem

4 Let $\ell$ be the total number of iterations of Step 2 of Algorithm $\mathcal {A}$. If the solution returned by the algorithm covers all vertices, then the solution is optimal and we are done. Otherwise, the solution returned by the algorithm may not be optimal, in which case it holds that $T_{\ell }$ violates the budget. We apply Lemma 7 to iteration $\ell$ in order to derive

$$\begin{aligned} p(T_{\ell })\ge & p(T^*)\left( 1 - \prod _{k' = 1}^\ell \left( 1- \frac{\alpha c(e_{k'})}{B}\right) \right) \\\ge & p(T^*)\left( 1 - \prod _{k' = 1}^\ell \left( 1- \frac{\alpha c(e_{k'})}{\sum _{e \in T_{\ell }} c(e)}\right) \right) \\\ge & p(T^*)\left( 1 - \prod _{k' = 1}^\ell \left( 1- \frac{\alpha \sum _{e \in T_{\ell }} c(e) / \ell }{\sum _{e \in T_{\ell }} c(e)}\right) \right) \\= & p(T^*)\left( 1 - \left( 1- \frac{\alpha }{\ell }\right) ^\ell \right) \\\ge & p(T^*)\left( 1-\frac{1}{e^{\alpha }}\right) , \end{aligned}$$

where the second inequality follows from the fact that $T_{\ell }$ violates the budget, and the third inequality follows from Lemma 8.

The algorithm outputs a set with a profit of $\max \{p(T_{\ell -1}),p(e_{\ell })\}$ and from the above derivation in follows that,

$$\begin{aligned} \max \{p(T_{\ell -1}),p(e_{\ell })\} \ge \frac{1}{2} (p(T_{\ell -1}) + p(\{e_{\ell }\})) \ge \frac{1}{2} p(T_{\ell }) \ge p(T^*)\frac{1-1/e^{\alpha }}{2}, \end{aligned}$$

which proves the claim. $\square$

### Proof of Theorem 3

With Algorithm $\mathcal {A}$ in hand, we now show how to reduce an instance *I* of GBMC to an instance *r*(*I*) of budgeted maximum coverage on the same set of vertices, such that the optimal solution of *r*(*I*) has the same profit as the optimal solution of *I*. The instance *r*(*I*) may have a superpolynomial number of hyperedges. However, instead of generating the budgeted maximum coverage instance explicitly, we construct only a 1/2-approximate cost-efficiency oracle $f_{r(I)}$ for *r*(*I*). We then use Algorithm $\mathcal {A}$ on $f_{r(I)}$ in order to obtain a $(1 - 1/\sqrt{e})/2$-approximately optimal solution to *r*(*I*) in polynomial time. Lastly, we show how to transform in polynomial time a feasible solution for *r*(*I*) into a feasible solution for *I* with equal profit. We begin by defining our reduction *r*.

#### Definition 2

Let $I = (G = (V,E), c, p,B)$ be an instance of GBMC where *G* is a graph. Define the budgeted maximum coverage instance *r*(*I*) as $r(I) = (G' = (V,E'), c', p,B)$, where

$$\begin{aligned} E' = \bigcup _{i \in V} E_i' \qquad \text { and } \qquad E_i' = \{S \cup \{i\}\ |\ \forall i' \in S : \{i',i\} \in E\}, \end{aligned}$$

and $c'$ assigns a cost to each hyperedge: For hyperedge $e \in E'$ we set $c'(e) = \sum _{i \in e} c(i)$.

In the above definition, $E_i'$ consists of the hyperedges *X* such that $i \in X$ and all other vertices in *X* are connected to *i* by an edge. In other words, $E'$ are all hyperedges such that the corresponding edges in *G* form a star. Note that *c* is a function that assigns a cost to each *vertex*, while $c'$ is a function that assigns a cost to each *hyperedge in* $E'$. Note that the vertex sets, profit functions, and budgets of *I* and *r*(*I*) are equal.

We first show that every feasible solution $X'$ for *r*(*I*) can be transformed into a feasible solution *X* for *I* in polynomial time such that the profit is preserved. Consider the following function $g_I$ that maps solutions of *r*(*I*) to *I*:

#### Definition 3

Let $I = (G = (V,E), c, p,B)$ be an instance of GBMC and let $X'$ be a feasible solution for $r(I) = (G' = (V,E'), c',p,B)$. The function $g_I$ maps $X'$ to the following solution for *I*.

$$\begin{aligned} g_I(X') = \{\{i',i\}\ \in E\ |\ \{i',i\} \in \cup X'\}. \end{aligned}$$

In words, in the above definition $g_I(X')$ is the set of edges of *G* that are contained in a hyperedge of $X'$.

#### Lemma 9

Let $X'$ be a feasible solution for *r*(*I*). The edge set $g_I(X')$ is computable in time $\mathcal {O}(mn \cdot |X'|)$. Moreover, the solution $g_I(X')$ is feasible (i.e., the total cost of all vertices covered by $g_I(X')$ does not exceed *B*). Also, $p(X') = p(g_I(X'))$.

#### Proof

For the first claim, observe that for each hyperedge in $X'$ and edge in *E* we need to check if that edge is contained in the hyperedge. This can be done in $\mathcal {O}(n)$ time.

The second claim follows from the fact that the edge set $g_I(X')$ covers the same vertex set as $X'$, and by definition

$$\begin{aligned} B \ge \sum _{e \in X'} c'(e) = \sum _{i \in \cup X'} c(i) \cdot |\{e \in X' : i \in e\}| \ge \sum _{i \in \cup X'} c(i) = \sum _{i \in \cup X'} c(i). \end{aligned}$$

The third claim follows from the fact that the edge set $g_I(X')$ covers the same vertex set as $X'$. $\square$

Next, we show that the optimal solution for *I* is at most the profit of the optimal solution for *r*(*I*). (Combined with the previous lemma, this entails that the optimal profits of *I* and *r*(*I*) are equal.)

#### Lemma 10

Let $p_{\text {opt}}$ be the maximum profit achievable in instance *I*. There exists a solution for *r*(*I*) with profit $p_{\text {opt}}$.

#### Proof

Let *X* be a profit-maximizing feasible solution for *I*. Assume without loss of generality that all paths in *G*[*X*] are of size at most 2. In other words: no edge in *X* covers two vertices that are both covered by another edge (such an edge can be removed from *X* without decreasing the profit). Under this assumption, all connected components of *G*[*X*] are stars. We construct from *X* a feasible solution $X'$ for *r*(*I*) that has the same profit, as follows. We define $X'$ to be the collection of hyperedges that each correspond to a connected component of *X*, i.e., for each maximal star of *X*, we add to $X'$ the hyperedge consisting of the vertices covered by that star.

Since no pair of hyperedges in $X'$ intersects, by definition of $c'$ the total cost $\sum _{e \in X'} c'(e)$ equals $\sum _{i \in \cup X} c(i) < B$, and therefore $X'$ is a feasible solution for *r*(*I*). Moreover, $X'$ and *X* cover the same set of vertices, and therefore the profit of *X* in *I* equals the profit of $X'$ in *r*(*I*). $\square$

A final ingredient that we need is a 1/2-approximate cost-efficiency oracle *f* for *r*(*I*).

#### Definition 4

We define the function *f* algorithmically as follows. Let *S* be the input argument to *f* (so *S* represents the vertex set already covered during the execution of algorithm $\mathcal {A}$.) Function *f* computes for each $i \in S$ a vertex set $e_i$ in the star centered at *i*, where $e_i$ is a budget-feasible substar that approximately maximizes the cost-efficiency. We output the set in $\{e_i : i \in V\}$ with the highest cost-efficiency. Concretely, *f* maps to the output of the following algorithm.

1. Let $V' \subseteq V$ be the vertices with a neighbor not in *S* (note that *i* itself may be in *S*). For each $i \in V'$:
  1. Initialize $e_i := \{i\}$, and $d_i = c(i)$. If $i \in S$, set $n_i := 0$, and otherwise set $n_i := p(i)$.
  2. Order non-increasingly by ratio $p(i')/c(i')$ the vertices $i' \in V \setminus S$ that are attached to *i* by a hyperedge in *G*. Denote this ordering by $\sigma _i$.
  3. For each $i' \in \sigma _i$, if the total cost of $e_i$ is at most *B* and if $(n_i + p(i'))/(d_i + c(i')) \ge n_i/d_i$, then add $i'$ to $e_i$, set $n_i := n_i + p(i')$, and set $d_i := d_i + c(i')$.
  4. If the total cost of $e_i$ exceeds *B*: Let $i'$ be the vertex in $e_i$ with the minimum $p(i')/c(i')$). We consider two substars of $e_i$ with total cost at most *B*: The set $\{i, i'\}$, and the set $e_i \setminus \{i'\}$. We set $e_i$ to be the substar with highest cost-efficiency among these two. Formally:
    1. If $i \not \in S$: if $(p(i) + p(i'))/(c(i) + c(i')) \ge (n_i - p(i'))/(d_i - p(i'))$ then set $e_i = \{i,i'\}$, $n_i := p(i)+p(i')$, and $d_i := c(i) + c(i')$. Otherwise set $e_i := e_i \setminus \{i'\}$, $n_i := n_i - p(i')$, and $d_i := d_i - c(i')$.
    2. If $i \in S$: if $p(i')/(c(i) + c(i')) \ge (n_i - p(i'))/(d_i - p(i'))$ then set $e_i := \{i,i'\}$, $n_i := p(i')$, and $d_i := c(i) + c(i')$ otherwise set $e_i := e_i \setminus \{i'\}$, $n_i := n_i - p(i')$, and $d_i := d_i - c(i')$.
2. Output the set in $\{e_i : |e_i| \ge 2 \wedge i \in V'\}$ with the highest cost-efficiency (i.e., the ratio $n_i/d_i$).

#### Lemma 11

The function *f* is a 1/2-approximate cost-efficiency oracle for *r*(*I*) and can be computed in $\mathcal {O}(n^2)$ time.

#### Proof

It is easy to see that the set output by *f* is always a hyperedge in $E'$, as it only outputs sets of hyperedges that correspond to stars of *G*. Moreover, in the last step, it is easy to verify that the set $\{e_i : |e_i| \ge 2 \wedge i \in V'\}$ is never empty when $S \not = V$. This implies that *f* is a valid cost-efficiency oracle. From the description of the algorithm above, it is also straightforward to see that *f* runs in time $\mathcal {O}(n^2)$: For each vertex, all neighbors are considered, where processing each neighbor takes a constant amount of time. (Here, we are not taking into account the bit-complexity of the arithmetic operations, although the runtime would remain polynomial if we take this aspect into account.)

What still needs to be proved is the approximation factor. Let $e_1, e_2, \ldots$ be the sets used in Step 2 of the algorithm. It suffices to show that for each $i \in V'$ for which it holds that $|e_i| \ge 2$, the ratio $n_i/d_i$ is at least $(1/2) \cdot \sum _{i \in e' \setminus S} p(i)/c(e')$ for all $e' \in E_i'$. In words, the cost-efficiency $n_i/d_i$ of the set $e_i$ is at least half the maximum cost-efficiency among all hyperedges in $E_i'$ (with respect to the input set *S*). (Note that we need not consider those $i \in V'$ for which $|e_i| = 1$: It can be easily verified that in this case, the optimal star centered at *i* is a single edge $\{i,i'\}$. This edge is also in $E_{i'}'$, and it is necessarily true that $|e_{i'}| \ge 2$.)

Let $i \in V'$ such that $|e_i| \ge 2$. Denote by $\Gamma (i)$ the vertices attached to *i* that are not in *S*. We will compare $|e_i|$ to an optimal *fractional* solution *x*: In this fractional solution each of the vertices $i'$ attached to *i* (and not in *S*) is picked with a certain fraction $x_{i'} \in [0,1]$, and vertex *i* is selected with fraction $x_i = 1$. The cost-efficiency is defined as

$$\begin{aligned} \frac{p(i) + \sum _{i' \in \Gamma (S)} x_{i'}p(i') }{\sum _{i' \in \Gamma (S)} x_i'c(i') } \end{aligned}$$

if $i \not \in S$, and otherwise as

$$\begin{aligned} \frac{\sum _{i' \in \Gamma (S)} x_{i'}p(i') }{\sum _{i' \in \Gamma (S)} x_i'c(i') }. \end{aligned}$$

Then it holds that the cost-efficiency of $e_i^{\text {frac}}$ exceeds the cost-efficiency of the hyperedge $e_i^* \in E_i'$ that maximizes $\sum _{i \in e^* \setminus S} p(i)/c(e^*)$, which would be the optimal integral solution.

We now consider the a greedy procedure for obtaining a fractional solution, and show that this particular procedure results in the optimal fractional solution *x*: $\square$

#### Claim 12

Consider the *greedy process* that selects vertices in $\Gamma (i)$ according to non-increasing cost-efficiency (i.e., according to the order $\sigma _i$ as given in Definition 4). If including a considered vertex increases the cost-efficiency of the solution, it is included with the highest possible fraction in [0, 1] such that the budget is not exceeded. Running the greedy process results in the optimal fractional solution *x*.

#### Proof

Note that, following the outlined greedy process, all vertices of $\Gamma (i)$ are selected with either fraction 0 or 1, except at most one vertex, which is selected with a fraction in (0, 1). To see why the claim is true, suppose for contradiction that *x* has a different structure. In that case, if there is a vertex $i' \in \Gamma (i)$ with $x_{i'}> 0$ such that the cost-efficiency of $i'$ is less than the cost-efficiency of *x*, then setting $x_{i'}$ to 0 will increase the cost-efficiency of the solution. (The latter follows by the simple fact that for two rationals *a*/*b* and *c*/*d* it holds that $(a+c)/(b+d) < a/b$ if and only if $c/d < a/b$.) Therefore, we may assume that the only vertices that are selected with a positive fraction, are vertices that have a cost-efficiency of at least the cost-efficiency of *x*. We can then consider the following operation: There must be two vertices $i', i'' \in \Gamma (i)$ for which it holds that $x_{i'} < 1$, $x_{i''}> 0$, and the cost-efficiency of $i'$ exceeds that of $i''$. In that case, decreasing $x_{i''}$ by an amount $\epsilon$ and increasing $x_{i'}$ by a maximal amount would increase the cost-efficiency (for a suitably small choice of $\epsilon$), which is a contradiction to *x* being optimal. This shows that *x* is obtained by the aforementioned greedy procedure.

Next, we observe that if *x* happens to be integral, then the set of integrally selected vertices is precisely $e_i$, which means that $e_i$ is the vertex set that maximizes the cost-efficiency. In this case the claim is proved. We now consider the case that *x* is not integral. From now on, let $i'$ be the vertex that is fractionally selected in *x* and let $S'$ be the integral vertices of *x* excluding *i*, i.e., $S' = \{i'' : i'' \not = i \wedge x_{i''} = 1\}$. It follows from Definition 4 that $e_i$ is either the set $\{i\} \cup S$ or the set $\{i, i'\}$. We distinguish four (very similar) cases.

- We first consider the case that $i \not \in S$ and $p(i') \ge \sum _{i'' \in S'} p(i'')$. Because *x* is the optimal fractional solution, the cost-efficiency of $S' \cup \{i,i'\}$ exceeds the optimal fractional solution and thus also the cost-efficiency of the optimal hyperedge $e_i^*$. Therefore, we conclude that the cost-efficiency of $e_i$ is at least
  $$\begin{aligned}&\frac{p(i) + p(i')}{c(i) + c(i')} \ge \frac{p(i) + p(i')}{c(i) + c(i') + \sum _{i'' \in S'} c(i'')} \ge \frac{1}{2} \cdot \frac{p(i) + p(i') + \sum _{i'' \in S'} p(i'')}{c(i) + c(i') + \sum _{i'' \in S'} c(i'')} \\&\qquad \ge \frac{1}{2} \cdot \frac{\sum _{i'' \in e_i^*} p(i)}{\sum _{i'' \in e_i^*} c(i)}, \end{aligned}$$
  as needed.
- In case $i \not \in S$ and $p(i') < \sum _{i'' \in S'} p(i'')$ we similarly obtain that the cost-efficiency of $e_i$ is at least
  $$\begin{aligned}&\frac{p(i) + \sum _{i'' \in S'} p(i'')}{c(i) + \sum _{i'' \in S'} c(i'')} \ge \frac{p(i) + \sum _{i'' \in S'} p(i'')}{c(i) + c(i') + \sum _{i'' \in S'} c(i'')}> \frac{1}{2} \cdot \frac{p(i) + p(i') + \sum _{i'' \in S'} c(i'')}{c(i) + c(i') + \sum _{i'' \in S'} c(i'')} \\&\qquad \ge \frac{1}{2} \cdot \frac{\sum _{i'' \in e_i^*} p(i)}{\sum _{i'' \in e_i^*} c(i)}. \end{aligned}$$
- The remaining two cases are:
  - the case that $i \in S$ and $p(i') \ge \sum _{i'' \in S'} p(i'')$;
  - the case that $i \in S$ and $p(i') < \sum _{i'' \in S'} p(i'')$.
  The analyses of those two cases are entirely analogous to those of the first two cases, where we simply replace *p*(*i*) with 0.

We are now ready to present the algorithm for GBMC on graphs, which we refer to as Algorithm $\mathcal {B}$. The algorithm is defined as follows. Let $I = (G = (V,E), c, p, B)$ be an input instance of GBMC where *G* is a graph.

- Run algorithm $\mathcal {A}$ on the 1/2-approximate cost-efficiency oracle *f* of Definition 4. This results in a solution $X'$ for instance *r*(*I*) (where *r*(*I*) is given in Definition 2).
- Compute and output $g_I(X')$ (see Definition 3).

The correctness, polynomial runtime, and approximation factor of $(1 - 1/\sqrt{e})/2$ of algorithm $\mathcal {B}$ follow directly from the lemmas and definitions above. Note that the bound on the runtime can most likely be improved by a more careful analysis, but that is beyond the scope and goal of this work.

$$\square$$

## GBMC when the incidence graph is a forest

In this section, we derive a bi-level dynamic program for the case when the incidence graph is a forest. We refer to this special case as GBMC-Forest. We also show that our dynamic program can be turned into an FPTAS, for which we introduce *P* as the maximum profit of a vertex, i.e., $P = \max _{v \in V} p(v)$.

### Theorem 13

GBMC-Forest can be solved optimally in time $\mathcal {O}(m n^3 P^2)$.

Suppose the given incidence graph is a forest and consists of *z* trees $T_1, \dots , T_z$. In order to facilitate the exposition of our dynamic program we combine these trees simply into a single tree *T* as follows. Introduce an artificial hyperedge $e_0$, representing the root of the tree. Furthermore, we introduce for each tree $T_t$ with $t \in [z]$ a dummy vertex node $d_t$ with zero profit and cost, i.e., $p(d_t) = c(d_t) = 0$, and connect it to $e_0$. Finally, we connect $d_t$ to its respective tree $T_t$ by adding an edge in the incidence graph from $d_t$ to an arbitrary hyperedge from $T_t$.

**Fig. 2:**
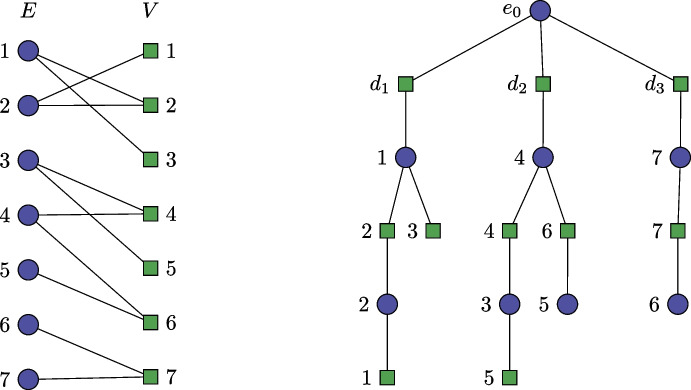
Example of an incidence graph (left) and its “unfolded’ tree (right)

This yields a bipartite graph that is a single tree. Note that the nodes along a path from the root to any other node are alternately hyperedge/vertex nodes. Assume we “unfold” this tree in order to draw the bipartite graph in a layered manner, as illustrated in Fig. 2.

Our dynamic program processes the unfolded tree *T* in a bottom-up manner. It consists of two separate dynamic programs, one for the hyperedges, and one for the vertices. We describe these programs informally in this section, and provide the technical details and proof of the dynamic program in Appendix C.

Consider an arbitrary subtree in *T* rooted at either a node represented by a hyperedge or vertex. Both dynamic programs rely on the fact that a subset of this subtree can be solved to optimality, which immediately can be used to solve a greater subset to optimality. In case the subtree is rooted at a node represented by a hyperedge, we consider the subtree up until the first *s* children in the subtree of the hyperedge. Once the optimal solutions (minimum required cost to obtain a specific profit, if possible) are known for every possible profit (upper bounded by *nP*), it is possible to find optimal solutions for the subtree until the first $s + 1$ children by linear enumeration. A similar, but slightly adapted method works in case the subtree is rooted by a vertex rather than a hyperedge. For the complete proof of Theorem 13 and technical details regarding the dynamic program, we refer to Appendix C.

Furthermore, we can employ standard techniques (profit truncation) to turn the above pseudo-polynomial time algorithm into an FPTAS, i.e., an algorithm that takes an error parameter $\varepsilon> 0$ and computes in time polynomial in the input size and $1 / \varepsilon$ a $(1 - \varepsilon )$-approximation to the optimal solution. The proof of the following theorem is given in Appendix D.

### Theorem 14

There exists an FPTAS for GBMC-Forest that runs in time $\mathcal {O}(m n^5 / \epsilon ^2)$.

## GBMC with a bounded size feedback vertex set

We have shown in the previous section that GBMC-Forest can be solved in pseudo-polynomial time and that there is an FPTAS. This implies that the inapproximability of the general problem is caused by the cycles in the incidence graph. In this section, we provide a fixed parameter tractability result that allows us to handle incidence graphs with cycles. The fixed parameter is the minimum number $\alpha$ of hyperedge nodes that we need to remove in order to make the incidence graph acyclic.

To provide an initial intuition, construct a graph $G' = (E, E')$ where every hyperedge $e \in E$ is represented by a node, and two nodes are connected if and only if the corresponding hyperedges share at least one element. This defines the edge set $E'$ in the new graph. Consider the case in which *G* contains solely one cycle. Select a hyperedge node of the cycle and fix whether this hyperedge is chosen or not in a solution. We then consider the reduced problem in which hyperedge *e* is removed. The incidence graph of the reduced problem is a forest and can thus be solved optimally by using the pseudo-polynomial time algorithm (or approximately by using the FPTAS) of the previous section. We may thus solve two variants of the instance in pseudo-polynomial time: In one variant, the hyperedge *e* is included in the solution, whereas in the other variant it is not. Under either of these assumptions, the instance reduces to a forest. Thus, if the graph contains one cycle, the running time is multiplied by 2 with respect to our pseudo-polynomial time algorithm for forests. We can extend this idea to more general graphs.

Define $\alpha$ as the minimum number of hyperedges whose removal turn the graph into a forest, i.e., $\alpha$ is the cardinality of the *minimum feedback vertex subset* of the hyperedge nodes of the incidence graph. We refer to the latter as the *minimum feedback hyperedge node set*. Then, the running time of the algorithms mentioned in the previous sections is multiplied by a factor of $2^\alpha$, because it is necessary to solve our problem on a forest for every subset of the $\alpha$ hyperedges.

The problem of finding a minimum feedback vertex set is $\textsf{NP}$-hard in general, but it is fixed parameter tractable. Kociumaka & Pilipczuk [13] give a deterministic $\mathcal {O}^*(3.62^\alpha )$ time algorithm to solve the problem, and Li & Nederlof [14] present a randomized $\mathcal {O}^*(2.7^\alpha )$ time algorithm, where the $\mathcal {O}^*$ notation is used to suppress factors that are polynomial in the input size.

### Theorem 15

There exists a deterministic algorithm for GBMC that runs in $\mathcal {O}^* (P^2 2^{\alpha } + 3.62^\alpha )$ time and a randomized algorithm for GBMC that runs in $\mathcal {O}^*(P^2 2^{\alpha } + 2.7^\alpha )$ time, where $\alpha$ is the size of the minimum feedback hyperedge node set.

All that needs to be shown is how to use the $\mathcal {O}^*(3.62^\alpha )$ time algorithm of [13] or the $\mathcal {O}^*(2.7^\alpha )$ time algorithm of [14], in order to find a minimum feedback vertex set restricted to only the hyperedge nodes of the incidence graph. This is straightforward: We reduce the incidence graph of *G* to the aforementioned multigraph $G' = (E, E')$ with only *E* as its vertex set. The edge set $E'$ is constructed as follows: there exists an edge between two hyperedges if they share at least one vertex in the original graph. It is now easy to see that there is a one-to-one correspondence between the cycles in the incidence graph of *G* and the cycles in $G'$, and each cycle in the incidence graph of *G* corresponds to a cycle in $G'$ on the same set of vertices. Therefore, a minimum feedback vertex set of $G'$ corresponds to a minimum feedback hyperedge node set of *G*, and the algorithms of [13] and [14] will find such a set deterministically in $\mathcal {O}^*(3.62^\alpha )$ time or randomized in $\mathcal {O}^*(2.7^\alpha )$ time, respectively.

**Fig. 3:**
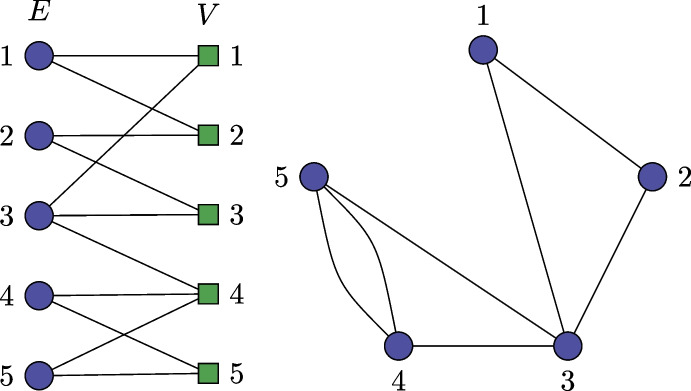
Example of a transformation to the feedback vertex set problem

The transformation is illustrated in Fig. 3. There, Hyperedges 3 and 5 are connected by Vertex 4 in (the incidence graph representation of) *G*, thus an edge $\{3, 5\}$ is included in $G'$. The edge labels are omitted. Note that Hyperedges 4 and 5 are connected by two edges, because they are both connected to Vertex 4 and Vertex 5.

## GBMC with bounded degree vertices

In this section we derive an approximation algorithm for GBMC for arbitrary hypergraphs $G = (V, E)$. Our algorithm has an approximation ratio of $\mathcal {O}(1/d^2)$, where *d* is the maximum degree of a node in the hypergraph, i.e. $d = \max \text {deg}(v) = \max \{|\{e : v \in e\}| : v \in V\}$. Our algorithm, which we name Algorithm $\mathcal {C}$, proceeds in three steps:

***Step 1: Duplication of vertices.*** Let $I = (G = (V, E), p, c, B)$ be an instance of GBMC. We define first a new instance obtained from *G* by duplicating each node a number of times equal to its degree, such that each hyperedge is attached to a unique copy of each of the nodes. Formally, we let $I' = (G' = (V',E'), p', c', B')$ where $V' = \{v_e\ :\ v \in V, e \in E, v \in E\}$ (hence, for each $v \in V$ there are $\text {deg}(v)$ vertices in $V'$ that correspond to *v*). and $E' = \{ \{v_e\ : v \in e\}\ : e \in E\}$. Furthermore, the cost and profit functions are defined such that they correspond to the costs and profits of the original instance: $c'(v_e) = c(v)$ and $p'(v_e) = p(v)$ for all $v \in V$ and $e \in E$ such that $v \in e$. Lastly, we set $B' = dB$.

***Step 2: Bin-packing the optimal solution of the decomposed instance.*** The instance $I'$ is trivially a forest, hence we can compute for any constant $\varepsilon \in (0,1)$ a $(1-\varepsilon )$-approximate solution $X'$ for $I'$ which respects the overall budget $B' = dB$, given the algorithm Section 3 (which for this particular case comes down to solving a simple knapsack instance).

The next step is to partition the edge set $X'$ into at most 2*d* sets $X_1', \ldots , X_{2d}'$ such that the total cost in each set does not exceed *B*. This is in essence a bin-packing problem (i.e., packing a set of items of varying weights in a set of bins of limited capacity). Because every edge induces cost at most *B* and the total cost is at most *dB*, standard bin-packing arguments show that such a partition exists and can be computed in polynomial time [15].^2^

***Step 3: Obtaining a solution for the original instance.*** Let $X^*$ be a set of maximum profit among the sets $X_1', \ldots , X_{2d}'$. We obtain a solution *X* for *I* from the solution $X^*$ by picking the hyperedges in *E* that naturally correspond to the hyperedges in $X^*$: Concretely, $X = \{\{v\ :\ v_e \in e\}\ :\ e \in X^*\}$. The algorithm outputs *X*.

### Theorem 16

Algorithm $\mathcal {C}$ is a $(1-\varepsilon )/(2d^2)$-approximation algorithm for GBMC that runs in polynomial time, where *d* is the maximum degree of a node.

### Proof

It is clear that the algorithm runs in polynomial time. Moreover, the algorithm outputs a feasible solution because the total cost induced by the nodes covered by $X^*$, with respect to $I'$ is at most *B* (by construction). Therefore, the total cost of *X* with respect to *I* is also at most *B*.

It remains to analyze the approximation ratio. Let $OPT _I$ be the optimal profit of the original instance *I* and let $OPT _{I'}$ be the optimal profit of $I'$. Note that any feasible solution for *I* corresponds directly to a feasible solution for $I'$, because the total cost of a solution for *I* is at most *d* times larger in $I'$ and $B' = dB$. Therefore, the total profit $p_{I'}(X')$ of $X'$ in $I'$ is at least $(1 - \varepsilon ) OPT _{I'} \ge (1 - \varepsilon ) OPT _I$.

Because for $X^*$ we choose the maximum-profit set among a partition of $X'$ consisting of 2*d* sets, the total profit of $X^*$ in $I'$ is at least $(1 - \varepsilon ) OPT _I/(2d)$. Also, the total profit of *X* in *I* is at most a factor *d* less than the total profit it induces in $I'$. Therefore, the total profit $p_I(X)$ of *X* in *I* satisfies $p_I(X) \ge (1 - \varepsilon ) OPT _I/(2d^2)$, which completes the proof. $\square$

## Discussion

In this paper we have presented various approximation algorithms for important special cases of the GBMC problem. With respect to our constant-factor approximation algorithm for the special case where the hypergraph is a graph, it is natural to ask the question whether a constant factor approximation is also possible to achieve for $\ell$-uniform hypergraphs, where $\ell$ is a constant. This is unfortunately unlikely, even for $\ell =3$, because the highly inapproximable densest *k*-subgraph problem [16] can be reduced to it. The following proof for this was provided to us by Chien-Chung Huang.

### Proposition 17

For the special case of the GBMC problem where the hypergraph is 3-uniform, there does not exist a polynomial time algorithm with an approximation factor better than $n^{1/(\log \log n)^c}$ for some constant *c*, unless the exponential time hypothesis is false.

### Proof

We present an approximation-factor-preserving reduction from the densest *k* subgraph problem, which is known to be inapproximable up to a factor $n^{1/(\log \log n)^c}$ (where $c> 0$ is a constant) conditional on the time hypothesis holding true [16]. An instance *I* of this problem is a graph *G*, and the task is to find a subset *S* with $|S| = k$ such that the number of edges of the subgraph *G*(*S*) of *G* induced by *S* is maximized.

Given *I* we create an instance $I' = (G'= (V',E'),c,p,B)$ of GBMC as follows. Let the hypergraph $G'$ have vertex set $V' = V \cup E$, and let $E' = \{\{e,u,v\}\ :\ e \in E, e = \{u,v\}\}$. We furthermore set $B = k$, and define *c* and *p* as follows. For $i \in V'$,

$$\begin{aligned} c(i) = {\left\{ \begin{array}{ll} 1 & \text { if } i \in V \\ 0 & \text { if } i \in E , \end{array}\right. } \end{aligned}$$

and

$$\begin{aligned} p(i) = {\left\{ \begin{array}{ll} 0 & \text { if } i \in V \\ 1 & \text { if } i \in E . \end{array}\right. } \end{aligned}$$

Let *S* be a set of *k* vertices of *G* and let *E*(*G*(*S*)) be the edge set of the induced subgraph *G*(*S*). Then in $I'$ it is a feasible solution to select the set of |*E*(*G*(*S*))| hyperedges $\{\{e,u,v\}\ :\ e \in E(G(S)), \{u,v\} \in e\}$, since there are only *k* vertices with cost 1 covered by this set. By the construction of *p* this set of hyperedges has a profit of |*E*(*G*(*S*))|.

Conversely, let $S'$ be a feasible subset of hyperedges of $G'$, i.e., $\sum _{i \in \cup S'} c(i) = \le B$. Then the set $S = S' \cap V$ has at most *k* vertices by the definition of *c*, so that *S* is a feasible solution of *I*. By construction $\sum _{i \in S'} p(i) = |S'| \le G(S)$. $\square$

Nonetheless, an interesting open problem would be to identify the best possible (non-constant) approximation factors that can be achieved in polynomial time for these special cases. We note that the best known polynomial time approximation factor for the densest *k*-subgraph problem is at present $n^{1/4 + \epsilon }$ [17].

One further special case of the GBMC problem we would like to mention is the one where we restrict the cost to be uniform across all vertices. This is a natural special case which we believe is interesting in its own right, and for which improved approximation factors could potentially be achieved.

## Figures and Tables

**Fig. 4:**
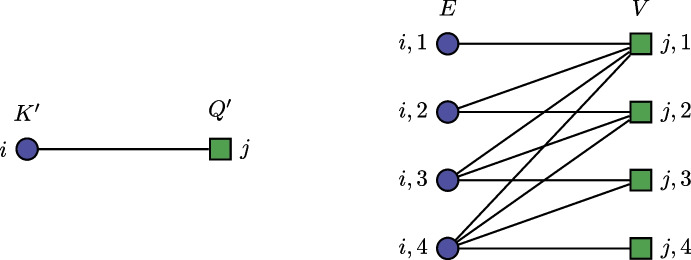
Example of an edge in they keyword-query graph transformed into hyperedges and vertices for the GBMC problem

**Fig. 5:**
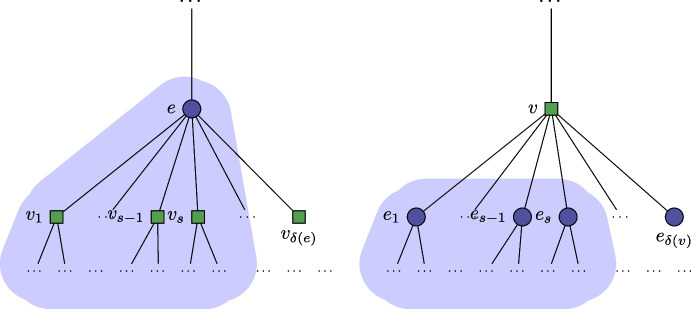
Illustration of the recurrence of the dynamic program for the hyperedges (left) and the vertices (right)

## Data Availability

No datasets were generated or analysed during the current study.

## Appendix A Proof of Theorem 1

Given an instance of Budgeted Bid Optimization, $G' = (K' \cup Q', E')$ with $|K'| = k$ and $|Q'| = q$, maximum bid *N*, and cost and profit functions $c_j'(n)$ and $p_j'(n)$, construct the following instance for GBMC (where for an integer $a \in \mathbb {N}$, we write [*a*] and $[a]_0$ to denote the sets $\{1, \ldots , a\}$ and $\{0, 1, \ldots , a\}$ respectively):

- $$V := \{v_{j,n}\ :\ j \in [q], n \in [N]\}$$.
- $$E := \{e_{i,n}\ :\ i \in [k], n \in [N]\}$$.
- $$e_{i, n} := \{v_{j, \ell } : \{i,j\} \in E', \ell \le n\}$$ for $$i \in k, n \in [N]$$.
- $$p_{j, n} := p_{j}' (n) - p_{j}' (n - 1)$$ for $$j \in [q], n \in [N]$$.
- $$c_{j, n} := c_{j} '(n) - c_{j}' (n - 1)$$ for $$j \in [q], n \in [N]$$.
- The budget remains *B*.

In words, for each keyword and each possible bid, a hyperedge is constructed. Furthermore, for each query and each possible bid, a vertex is constructed. A hyperedge is then connected to a vertex if the corresponding keyword and query form an edge of $G'$, and the corresponding bid of the hyperedge is higher than or equal to the corresponding bid on the vertex.

Furthermore, the profit $p_{(n - 1) N + j}$ of an element in the newly constructed instance of GBMC corresponds to the marginal profit of a query in Budgeted Bid Optimization. To be precise, $p_{j, n}$ corresponds to the marginal profit of query *j* when the bid would be increased from $n - 1$ to *n* (same holds for the costs). An example is shown in Fig. 4, where the maximum bid $N = 4$.

To see why this is an approximation-preserving reduction, one can observe that there exists the following mapping between solutions of the two instances, which preserves their respective costs and profits: Suppose that for our Budgeted Bid Optimization instance we have a solution $b_1, \dots , b_k$ ($b_i \in [N]$ for all keywords $i \in K$). For each bid $b_i$, we may select the $b_i$-th hyperedge corresponding to vertex *i*, i.e., vertex $v_{i, b_{i}}$. By construction, the costs and profits of the resulting solution for the GBMC instance are the same as our solution for the Budgeted Bid Optimization instance.

Conversely, let $e^* \subseteq E$ be a solution to our reduced-to $\text {GBMC}$ instance. Without loss of generality, one can assume that if $e_{i, n}$ is selected, then $e_{i, 1}, \dots , e_{i, n - 1}$ are not selected. Selecting $e_{i, n}$ corresponds exactly to the cost and profit when bidding *n* on keyword *i*. Doing so in our Budgeted Bid Optimization instance will yield the same costs and profits. This proves Theorem 1.

## Appendix B Proof of Lemmas 5 to 8

### Proof of Lemma

5 Let $e_1^*, \ldots , e_{|T^*|}^*$ be the hyperedges of $T^*$. Let $e_{\max }^*$ be the element of $T^*$ with the highest cost-efficiency when it would be added to the solution $T_k$. That is:

$$\begin{aligned} e_{\max }^* = \arg \max _{e \in T^*} \frac{\sum _{i \in e \setminus S_{k-1}} p(i)}{c(e)} . \end{aligned}$$

Note that the hyperedge $e_k$ selected by the algorithm in iteration *k* has a cost-efficiency that is within a factor of $\alpha$ from that of the hyperedge $e_{\max }^*$. Suppose now that we would add all of $T^*$ to $T_{k-1}$. We can bound the profit that we would gain from this as follows.

$$\begin{aligned} \sum _{i \in (\cup T^*) \setminus S_{k-1}} p(i)\le & \sum _{j = 1}^{|T^*|} \sum _{i \in e_j^* \setminus S_{k-1}} p(i) \\\le & \sum _{j = 1}^{|T^*|} c(e_j^*) \sum _{i \in e_j^* \setminus S_{k-1}} \frac{p(i)}{c(e_j^*)} \\\le & \sum _{j = 1}^{|T^*|} c(e_j^*) \sum _{i \in e_{\max }^* \setminus S_{k-1}} \frac{p(i)}{c(e_{\max }^*)} \\\le & B \sum _{i \in e_{\max }^* \setminus S_{k-1}} \frac{p(i)}{c(e_{\max }^*)} \\\le & B \sum _{i \in e_{k} \setminus S_{i-1}} \frac{p(i)}{\alpha c(e_{k})} \\= & \frac{B}{\alpha c(e_k)}(p(T_k) - p(T_{k-1})). \end{aligned}$$

### Proof of Lemma

6 We derive this as follows by applying Lemma 5.

$$\begin{aligned}&\frac{B}{\alpha c(e_k)}(p(T_k) - p(T_{k-1})) \ge \sum _{i \in (\cup T^*) \setminus S_{k-1}} p(i) \\&\qquad \ge \sum _{i \in (\cup T^*) \cap S_{k-1}} p(i) + \sum _{i \in (\cup T^*) \setminus S_{k-1}} p(i) - \sum _{i \in (\cup T^*) \cap S_{k-1}} p(i) - \sum _{i \in S_{k-1} \setminus (\cup T^*)} p(i) \\&\qquad = p(T^*) - p(T_{k-1}). \end{aligned}$$

### Proof of Lemma

7 We prove this by induction on the number of iterations *k*. If $k = 1$, we see that

$$\begin{aligned} p(T_k) = p(T_1) - p(T_0) \ge \frac{\alpha c(e_1)}{B} \sum _{i \in \cup T^*} p(i) = P(T^*) \left( 1 - \left( 1 - \frac{\alpha c(e_1)}{B}\right) \right) , \end{aligned}$$

where the inequality follows from Lemma 5. Suppose now that the claim holds if $k \in [\ell ]$ for some $\ell \ge 1$. We will prove that the claim also holds if $k = \ell +1$.

$$\begin{aligned}&p(T_{\ell +1}) = p(T_{\ell }) + p(T_{\ell +1}) - p(T_{\ell }) \\&\qquad \ge p(T_{\ell }) + \frac{\alpha c(e_{\ell + 1})}{B}(p(T^*) - p(T_{\ell })) \\&\qquad = \left( 1 - \frac{\alpha c(e_{\ell +1})}{B}\right) p(T_{\ell }) + \frac{\alpha c(e_{\ell + 1})}{B}p(T^*) \\&\qquad \ge p(T^*) \left( 1 - \frac{\alpha c(e_{\ell +1})}{B}\right) \left( 1 - \prod _{k' = 1}^{\ell } \left( 1 - \frac{\alpha c(e_{k'})}{B}\right) \right) + \frac{\alpha c(e_{\ell + 1})}{B}p(T^*) \\&\qquad = p(T^*) \left( 1 - \prod _{k' = 1}^{\ell +1} \left( 1 - \frac{\alpha c(e_i)}{B}\right) \right) , \end{aligned}$$

where the first inequality follows from Lemma 6 and the second inequality follows from the induction hypothesis.

### Proof of Lemma

8 We show that the function

$$\begin{aligned} g'(x) = 1 - g(x) = \prod _{i = 1}^n\left( 1 - \frac{x_i}{B}\right) \end{aligned}$$

on the domain *D* achieves its maximum when $x_i = a/n$ for all $i \in [n]$. Suppose we have a solution $x \in D$ for which there are two indices *i* and *j* such that $x_i> a/n$ and $x_j < a/n$. Assume without loss of generality that $i = 1$ and $j = 2$. Let $x'$ be the vector obtained from $x'$ by subtracting an amount of $\epsilon = (x_1 - x_2) / 2$ from $x_1$ and adding it to $x_2$. We show that $g'(x')> g'(x)$. Let $C = (1 - x_3/B) \cdots (1 - x_n/B)$. Then indeed,

$$\begin{aligned} g'(x')= & \left( 1 - \frac{x_1 - \epsilon }{B}\right) \left( 1 - \frac{x_2 + \epsilon }{B}\right) \prod _{i = 3}^n\left( 1 - \frac{x_i}{B}\right) \\= & g(x) + C \left( \frac{\epsilon }{B}\left( 1 - \frac{x_2}{B}\right) - \frac{\epsilon }{B}\left( 1 - \frac{x_1}{B}\right) - \frac{\epsilon ^2}{B^2} \right) \\= & g(x) + C \left( \frac{\epsilon x_1 - \epsilon x_2}{B^2} - \frac{\epsilon ^2}{B^2} \right) \\= & g(x) + C \left( \frac{(x_1 - x_2) x_1 - (x_1 - x_2) x_2}{2 B^2} - \frac{(x_1 - x_2)^2}{4B^2} \right) \\= & g(x) + C \left( \frac{(x_1 - x_2)^2}{4B^2} \right) \\> & g(x). \end{aligned}$$

This shows that a vector *x* does not maximize $g'$ whenever not all elements of *x* are equal, and thus establishes the claim.

## Appendix C Dynamic program for GBMC-Forest

In order to prove Theorem 3, we firstly provide several definitions. Given a hyperedge *e*, represented by a circled node in the tree *T*, let *T*(*e*) refer to the subtree of *T* rooted at *e*. Further, define $\delta (e)$ as the number of children of *e* in the tree *T*. For simplicity, we adopt the same notation *T*(*v*) and $\delta (v)$ for the subtree rooted at a vertex *v* and the number of children, respectively.

The dynamic program for the hyperedges is as follows: Fix a hyperedge $e \in E$ and denote its children (vertices) by $v_1, \dots , v_{\delta (e)}$. Define *T*(*e*, *s*) with $s \in [\delta (e)]$ as the subtree of *T*(*e*) induced by the union of *e* and the subtrees of the first *s* children $v_1, \dots , v_s$ of *e*. For every $s \in [\delta (e)]$, $x \in \{0, 1\}$, and $p \in [n P]_0$, we define

$$\begin{array}{ll} f (e, s, x, p) = & \text {minimum total cost incurred in the subtree}\,\, T(e, s)\,\, \text {such that the total}\\ & \text {profit is}\,\, p\,\, \text {and hyperedge}\,\, e\,\, \text {is selected in the solution}\,\, (x = 1)\\ & \text {or not}\,\, (x = 0). \end{array}$$

The dynamic program for the vertices is slightly more complex. Consider a vertex $v \in V$ and let its children (hyperedge) be denoted by $e_1, \dots , e_{\delta (v)}$. Define *T*(*v*, *s*) with $s \in [\delta (v)]$ as the collection of subtrees $T(e_1), \dots , T(e_s)$ (not including *v* itself). For every $s \in [\delta (v)]$, $x \in \{0, 1\}$ and $p \in [n P]_0$, we define

$$\begin{array}{ll} g (v, s, y, p)=& \text {minimum total cost incurred in}\,\, T(v, s)\,\, \text {such that the total profit is}\\ & \,\, p\,\, \text {and at least one of the children hyperedges}\,\, e_1, \dots , e_{s}\,\, \text {is selected}\\ & \text {in the solution}\,\, (y = 1)\,\, \text {or not}\,\, (y = 0). \end{array}$$

The parts/sets of the tree which the two dynamic programs consider, is visualized in Fig. 5. We process the unfolded tree in a bottom-up manner, computing $f$ and $g$ layer by layer, until eventually we have determined $f$ for the root node 0. The total profit of an optimal solution can then be determined as follows:

$$OPT = \max \{p \in [n P]_0 \; | \; f (0, \delta (0), 0, p) \le B\}.$$

We turn to the formal definitions of our dynamic programs. For a hyperedge $e \in E$, $s \in [\delta (e)]$, and $p \in [n P]_0$, we obtain the recurrence

$$\begin{aligned} f (e, s, x, p) = \min _{y \in \{0, 1\}, r \in [p]_0}&\big \{ f (e, s - 1, x, p - r - p(v_s) \cdot \max \{x, y\}) \\&\quad + g (v_s, \delta (v_s), y, r) + c(v_s) \cdot \max \{x, y\} \big \}. \end{aligned}$$

To see this, fix some $r \in [p]_0$. Then, depending on whether $v_s$ is chosen or not, the minimum cost to achieve profit *r* on the subtrees in $T(v_s, \delta (v_s))$ is either $g (v_s, \delta (v_s), 0, r)$ or $g (v_s, \delta (v_s), 1, r)$.

Further, $v_s$ itself contributes a cost of $c(v_s) \cdot \max \{x, y\}$ to the objective function, since the costs $c(v_s)$ needs to be only added if $x = 1$ (parent hyperedge of $v_s$ is chosen) or $y = 1$ (at least one child hyperedge of $v_s$ is chosen), or both. The minimum cost to obtain the remaining profit of $p - r - p(v_s) \cdot \max \{x, y\}$ on the subtree $T(e, s - 1)$ is $f (e, s - 1, x, p - r - p(v_s) \cdot \max \{x, y\}))$ by definition.

For a vertex $v \in V$, $s \in [\delta (v)]$, and $p \in [n P]_0$, we distinguish to cases. In case $y = 0$, obtain the a similar recurrence as for the hyperedges:

$$\begin{aligned} g (v, s, 0, p)&= \min _{r \in [p]_0} \big \{ g (v, s - 1, 0, p - r) + f (e_s, \delta (e_s), 0, r) \big \}. \end{aligned}$$

To see this, fix some $r \in [p]_0$. If $y = 0$, thus if all hyperedges $v_1, \dots , v_s$ are not selected in the solution. Basically, we need to consider the case where $v_1, \dots , v_{s - 1}$ are not selected in the optimal solution (first term), combined with the case where $v_s$ is also not selected (second term).

However, there are multiple possibilities to ensure that at least one child of *v* is selected in the optimal solution, i.e., to ensure $y = 1$. Either a child in $v_1, \dots , v_{s - 1}$ is selected while $v_s$ is not, or no child $v_1, \dots , v_{s - 1}$ is selected while $v_s$ is selected, or in both sets there exist selected children. These three cases are described in the dynamic program as follows:

$$\begin{aligned} g (v, s, 1, p)&= \min _{r \in [p]_0} \min \big \{ g (v, s - 1, 0, p - r) + f (e_s, \delta (e_s), 1, r), \\&\hspace{3em} \min _{x \in \{0, 1\}} g (v, s - 1, 1, p - r) + f (e_s, \delta (e_s), \min \{x, y\}, r) \big \}. \end{aligned}$$

Note that the last two out of three cases are combined through the last min-term in the above expression.

We next analyze the running time to compute these dynamic programs: Computing the value of $f (e, s, x, p)$ for fixed parameters takes time at most $\mathcal {O}(nP)$ (since $p \in [n P]_0$) and this has to be done at most $n^2 P$ times for every hyperedge; thus time $\mathcal {O}(m n^3 P^2)$ in total. Similarly, computing the value of $g (v, s, y, p)$ for fixed parameters takes time at most $\mathcal {O}(n P)$ and this has to be done *mnP* times for every vertex; thus time $\mathcal {O}(m n^3 P^2)$ in total.

## Appendix D FPTAS

Let $y_i = 1$ if vertex $v_i$ is selected in a solution, and $y_i = 0$ otherwise. The optimal selection of vertices to VWMC-Forest is then denoted by $y^* = \{y_1^*, \dots , y_m^*\}$. The idea is to truncate the values that the profit function takes on, i.e., for all $i \in [n]$:

$$\begin{aligned} p'(i) = \left\lfloor {\frac{p(i)}{10^t}}\right\rfloor , \end{aligned}$$

after which a solution is determined via dynamic programming. With these truncated profits, a solution can be determined in $\mathcal {O}(m n^3 (P/10^{t})^2)$ instead of $\mathcal {O}(m n^3 P^2)$. However, the optimal selection of vertices using the truncated profits, $y'$, could be suboptimal compared to the optimal selection $y^*$ with the original profit functions. Yet, a useful relationship holds between the two solutions, which we use in order to derive an FPTAS, namely,

$$\begin{aligned} \sum _{i = 1}^n p(i) \cdot y_i' \ge \sum _{i = 1}^n 10^t p'(i) \cdot y_i' \ge \sum _{i = 1}^n 10^t p'(i) \cdot y_i^* \end{aligned}$$

The first inequality follows directly from the definition of $p'(i)$, while the second inequality follows from the fact that $y'$ is optimal in the problem with truncated profits. Note that $y'$ corresponds to a feasible solution, since the cost functions remain the same. Furthermore, due to rounding down, it must be that $10^t p'(i) \cdot y_i^* \ge p(i) \cdot y_i^* - 10^t$. Hence, combining all mentioned (in)equalities, we obtain:

$$\begin{aligned} \sum _{i = 1}^n p(i) \cdot y_i^*&\ge \sum _{i = 1}^n (p(i) \cdot y_i^* - 10^t) = \sum _{i = 1}^n p(i) \cdot y_i^* - n \cdot 10^t \\&= OPT - n \cdot 10^t = OPT \left( 1 - \frac{n \cdot 10^t}{OPT}\right) \\&\ge OPT \left( 1 - \frac{n \cdot 10^t}{P}\right) , \end{aligned}$$

where the last inequality holds because $OPT \ge P$. Now fix $t = \log _{10} (\epsilon P/n)$ for some given $\epsilon> 0$, such that $n \cdot 10^t/P = \epsilon$. Then, by combining all mentioned (in)equalities, we conclude that

$$\sum _{i = 1}^n p(i) \cdot y_i' \ge (1 - \epsilon )OPT,$$

meaning that we have obtained an $(1 - \epsilon )$-approximate solution for VWMC-Forest. Since the running time of the dynamic program using truncated profits is $\mathcal {O}(m n^3 P^2 / 10^{2t})$ and because $n \cdot 10^t/P = \epsilon$, the running time of the new algorithm is $\mathcal {O}(m n^5 / \epsilon ^2)$, which directly results in Theorem 14.

## Footnotes

1 Throughout this paper, for a collection of sets *F* we write $\cup F$ to refer to the set $\cup _{S \in F} S$.

2 To clarify: We can view each edge as an item of weight equal to the cost induced by $X'$. The goal then is to pack these items into bins of capacity *B*.
